# Supplementary material for: Baru Almond Beverage (Baruccino) with Different Sweeteners: Nutritional and Physical Properties and Exploration of Sensory and Non-Sensory Perceptions
Source: Foods. 2026 Jan 1;15(1):127. doi: 10.3390/foods15010127 (PMC12786057; doi:10.3390/foods15010127)

Supplementary Materials Figure S1. Labels presented during sample evaluation, in the second session (informed test). **Source:** The authors.

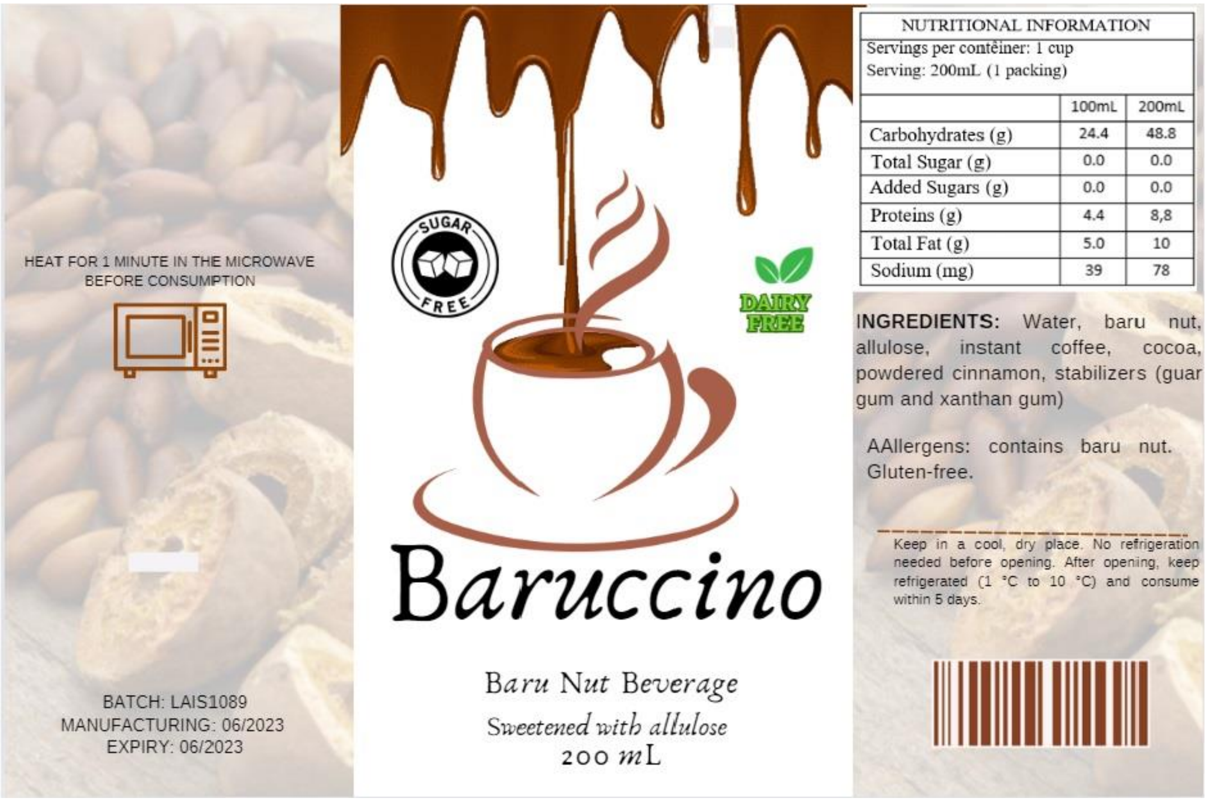

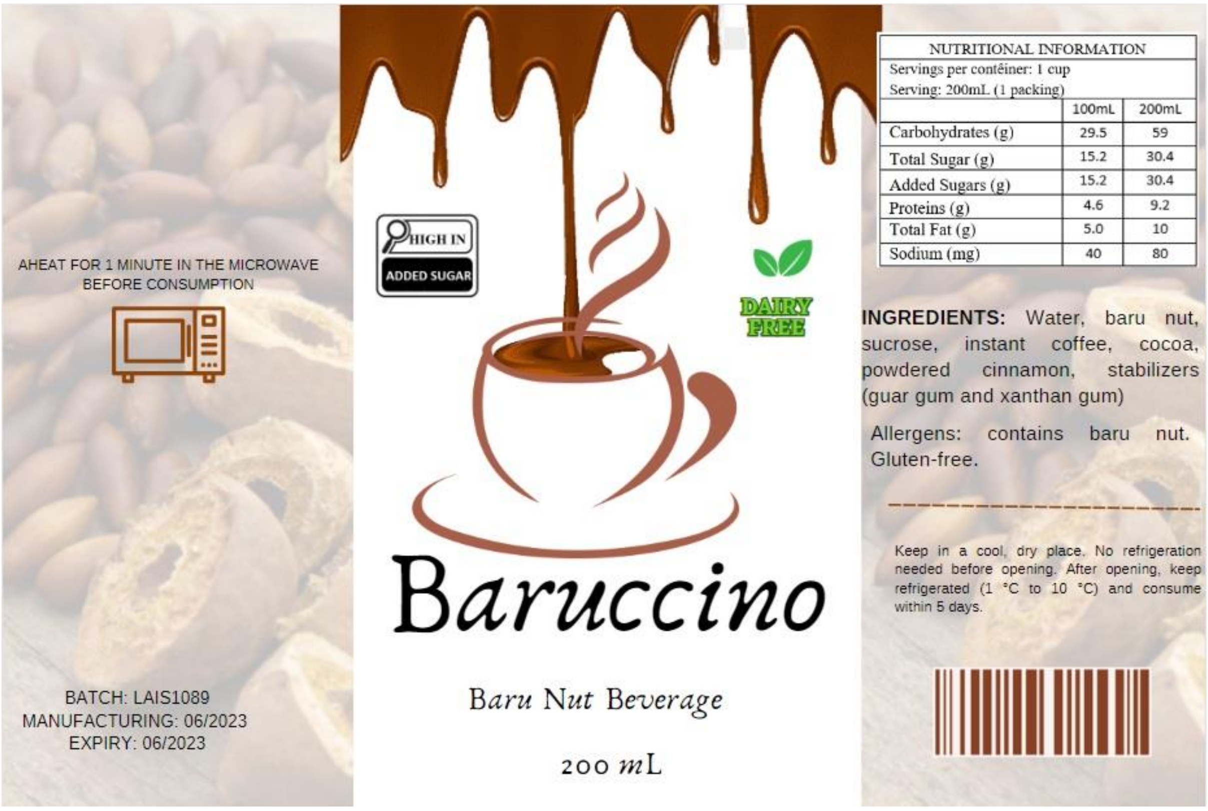

Supplement: Supplementary file 1 [file foods-15-00127-s001.zip › Figure S1- Supplementary material.pdf]
