# Supplementary material for: Baru Almond Beverage (Baruccino) with Different Sweeteners: Nutritional and Physical Properties and Exploration of Sensory and Non-Sensory Perceptions
Source: Foods. 2026 Jan 1;15(1):127. doi: 10.3390/foods15010127 (PMC12786057; doi:10.3390/foods15010127)
Supplement: Supplementary file 1 [file foods-15-00127-s001.zip › File S1- Supplementary material.pdf]

Supplementary Materials File S1 - Form used in sensory analysis sessions.

Name:____________________ Gender: F ( ) M ( ) Year of birth: _________
Date:____________Telephone______________________________Email:______________

Please evaluate the appearance and aroma of the sample, and then taste it. Identify (mark with an X) your opinion regarding the intensity of the attributes according to the scale below:

Sample code:___________

	ATTRIBUTES	Not Applicable	Low	Medium	High
Appearance	Brown color	( )	( )	( )	( )
	Brightness	( )	( )	( )	( )
Aroma	Cocoa aroma	( )	( )	( )	( )
	Chesnut aroma	( )	( )	( )	( )
Flavor	Sweet taste	( )	( )	( )	( )
	Cocoa flavor	( )	( )	( )	( )
	Pleasant flavor	( )	( )	( )	( )
	Chesnut flavor	( )	( )	( )	( )
	Cinnamon flavor	( )	( )	( )	( )
	Unpleasant flavor	( )	( )	( )	( )
	Fatty	( )	( )	( )	( )
Texture	Creamy	( )	( )	( )	( )
	Homogeneous texture	( )	( )	( )	( )
	Presence of particles	( )	( )	( )	( )

ACCEPTANCE

Please, rate using the scale below how much you liked or disliked the product in relation to the attributes:

9 - Like extremely
8 - Like very much
7 - Like moderately
6 - Like slightly
5 - Neither like nor dislike
4 - Disliked slightly
3 - Dislike moderately
2 - Dislike very much
1 - Dislike extremely

Appearance: ____________
Aroma: ______________
Texture: ______________
Flavor: _____________
Overall impression: ___________

Comments: ____

PURCHASE INTENTION

( ) Definitely would buy
( ) Probably would buy

( ) Might or might not buy
( ) Probably would not buy
( ) Definitely would not buy
Comments: ____

File S1. English version of the evaluation form model presented to consumers during the two sensory analysis sessions.
